# CpxA Deletion Is Associated with Altered Polymyxin B Susceptibility and Flagellar-Associated Transcriptional Remodeling in *Salmonella* Typhimurium

**DOI:** 10.3390/microorganisms14102237

**Published:** 2026-10-03

**Authors:** Yupeng Fang, Kuanjia Guo, Xiaoqing Zhang, Tiantian Chen, Chengbin Xue, Yangxinyue Zhan, Ziwei Guo, Cunlei Liu, Hao Lin, Yu Liu, Chimedtseren Bayasgalan, Shunli Yang, Yongsheng Liu

**Affiliations:** 1 Hebei Key Laboratory of Preventive Veterinary Medicine, College of Animal Science and Technology, Hebei Normal University of Science and Technology, Qinhuangdao 066004, China; 2 School of Veterinary Medicine, Mongolian University of Life Sciences, Ulaanbaatar 17024, Mongolia; chimedtseren.b@muls.edu.mn

**Keywords:** *Salmonella* Typhimurium, CpxAR two-component system, polymyxin B susceptibility, *cpxA*, flagellar assembly, envelope stress

## Abstract

Polymyxin B is considered a last-resort antibiotic for multidrug-resistant or extensively drug-resistant Gram-negative bacterial infections. Understanding the determinants of polymyxin B susceptibility in *Salmonella* is important for public health. Phenotypic analyses showed that deletion of *cpxA* was associated with altered stress tolerance and a one-dilution difference in the reported polymyxin B MIC, whereas the Δ*cpxA*Δ*arcB* double mutant exhibited broader growth and stress-related defects. Benjamini–Hochberg-controlled RNA sequencing under polymyxin B exposure identified 60 differentially expressed genes (35 with higher and 25 with lower expression in Δ*cpxA*), including 12 flagellar-associated genes and significant enrichment of flagellar functions. RT-qPCR showed significantly higher relative expression of four selected FDR-significant flagellar-associated genes in the Δ*cpxA* strain; *fliC*, *fliS*, *fliK*, and *flgE* were directionally concordant with RNA-seq, and expression returned toward WT-like levels in the complemented strain. Representative micrographs illustrated differences in extracellular filamentous appearance. These findings associate *cpxA* deletion with selective flagellar-associated transcriptional changes under polymyxin B exposure, accompanied by a one-dilution reported MIC difference and qualitative differences in extracellular filamentous appearance.

## 1. Introduction

*Salmonella enterica* serovars Typhimurium and Enteritidis are among the most common serovars associated with human salmonellosis [1]. Salmonellosis is a major challenge in animal production because it adversely affects animal health and productivity and causes substantial economic losses in the livestock industry [2]. Polymyxin B is a clinically available polymyxin used to treat infections caused by multidrug or extensively drug-resistant Gram-negative bacteria [3] and is considered a last-resort treatment for such infections [4]. Therefore, understanding the mechanisms that influence polymyxin B susceptibility in *Salmonella* is important for public health.

CpxAR (CpxA/CpxR) is a regulatory two-component system. In this system, CpxA is both a sensor kinase and a phosphatase of the cognate CpxR response regulator. Upon sensing extracytoplasmic stresses, phosphorylated CpxR activates or represses its target genes in bacteria [5], which can contribute to the stress response and virulence in various Gram-negative pathogens [6]. In many antibiotic-resistant strains, including *Enterobacter aerogenes* [7], *Achromobacter xylosoxidans* [8], *Salmonella enterica* serovar Paratyphi *A* [9], *Providencia rettgeri* [10], *Proteus mirabilis* [11] and *Serratia marcescens* [12], mutations in *cpxA* have been detected, suggesting a potential link between CpxA and bacterial antibiotic resistance. Previous studies have demonstrated that the CpxAR system influences diverse physiological and virulence-associated traits in Gram-negative bacteria. In *C. sakazakii*, CpxAR deletion strains showed weakened bacterial adhesion and invasion ability, lower levels of tight junction protein disruption, reduced apoptosis and cytotoxicity in cells cultured in vitro, as well as attenuated virulence in newborn mice [6]. In *Shigella*, the invasive phenotype can be repressed at low pH, and this pH-dependent regulation also requires CpxAR [13]. In enteropathogenic *Yersinia pseudotuberculosis*, CpxAR could influence biofilm development through its impact on exopolysaccharide production [14]. In *Actinobacillus pleuropneumoniae*, CpxAR plays an important role in cold resistance by upregulating *cspC* expression [15]. CpxAR contributes to biofilm formation in *A. pleuropneumoniae* [16]. In addition, *cpxAR* and *cpxD* mutants exhibited a non-capsulated phenotype and were strongly impaired in virulence in mice, suggesting that CpxAR plays an important role in capsule export and bacterial survival in the host [17]. In *Lonsdalea populi*, CpxAR could be involved in the regulation of virulence and reactive oxygen species resistance, and is crucial during infection and the stress response [18]. These findings suggest a key role for CpxAR in the development of bacterial virulence, as well as in the response to various environmental stresses.

The role of CpxAR in antimicrobial susceptibility has also received increasing attention. In *Klebsiella pneumoniae*, a CpxAR-deficient strain was more susceptible to β-lactams and chloramphenicol than the wild-type strain [19]. In a *Proteus mirabilis* clinical isolate, alterations in *cpxA* were associated with imipenem and amikacin resistance without a major growth defect [11]. CpxAR has also been implicated in multidrug resistance in *Salmonella enterica* serovar Enteritidis through effects on membrane permeability and efflux-pump activity [20]. In *S.* Typhimurium, *cpxR* influences susceptibility to aminoglycosides and β-lactams through regulation of multidrug-resistance-associated genes, including *acrB* [21], whereas deletion of *cpxA*/*cpxR* has been associated with reduced ceftriaxone resistance [22]. In *Y. pseudotuberculosis*, Δ*cpxA* mutants displayed increased sensitivity to colistin and polymyxin B in association with CpxA-dependent Lipid-A remodeling [23]. However, how CpxAR influences antimicrobial susceptibility across Gram-negative bacteria remains incompletely understood, and its role in polymyxin B susceptibility in *S.* Typhimurium has not been well characterized.

ArcB is the sensor kinase of the ArcAB two-component system, which coordinates central metabolism, respiratory adaptation, redox balance, and bacterial fitness [24]. To explore whether additional disruption of metabolic-redox regulation was associated with broader phenotypic changes following *cpxA* deletion, the Δ*cpxA*Δ*arcB* double mutant was also included as an exploratory extension of the phenotypic analyses.

In this study, we evaluated phenotypic, transcriptional, and morphological changes associated with *cpxA* deletion in *S. enterica* serovar Typhimurium under polymyxin B exposure. We further examined whether these transcriptional changes were accompanied by alterations in flagellar-associated gene expression and extracellular filamentous morphology.

## 2. Materials and Methods

### 2.1. Bacterial Strains and Plasmids

The λ-Red homologous recombination plasmids pKD46, pKD4, and pCP20, together with the shuttle plasmid pBR322, were maintained in our laboratory and used for strain construction and complementation. All *S. enterica* serovar Typhimurium strains, including SL1344, SL1344Δ*cpxA*, SL1344Δ*cpxA*/pBR322-*cpxA*, SL1344Δ*cpxA*Δ*arcB*, SL1344Δ*cpxA*Δ*arcB*/pBR322-*cpxA-arcB*, were validated via PCR or gene sequencing. The primers used for strain construction and verification are listed in Supplementary Table S1.

### 2.2. Growth Curve Analysis

Overnight cultures were diluted 1:100 in fresh LB broth and incubated at 37 °C with shaking at 180 rpm. Optical density at 600 nm was measured using a T2600 UV–visible spectrophotometer (Shanghai Yoke Instrument Co., Ltd., Shanghai, China) every 2 h.

### 2.3. Swimming Motility Assay

Bacterial cultures were adjusted to an OD600 of 1.0, washed with ice-cold PBS, and resuspended in PBS. A 2 μL aliquot was inoculated at the center of LB semisolid agar and incubated at 37 °C for 12 h. Swimming-zone diameters were measured and recorded.

### 2.4. Biofilm Formation Assay

Bacterial suspensions were adjusted to an OD600 of 0.2, and 200 μL was added to each well of a 96-well plate and incubated at 37 °C for 36 h. The wells were fixed with methanol for 15 min, washed with PBS, stained with 0.5% crystal violet for 30 min, and washed three times with PBS. Bound crystal violet was solubilized with 200 μL ethanol for 30 min, and absorbance was measured at 570 nm.

### 2.5. Environmental-Stress Assays

Overnight cultures were diluted 1:100 in LB broth under the indicated stress conditions. Acid stress was evaluated in medium adjusted to pH 4.0, hypertonic stress was evaluated in medium containing 2.5 M NaCl, and oxidative stress was evaluated in medium containing 10 mM H_2_O_2_. Cultures were incubated at 37 °C with shaking at 180 rpm for 3 h, serially diluted, and plated on LB agar. After overnight incubation, colony counts were determined. Relative survival was calculated by comparing colony counts obtained under each stress condition with those obtained for the corresponding strain under the non-stress condition.

### 2.6. Polymyxin B Susceptibility

A broth microdilution assay based on CLSI recommendations was used to determine polymyxin B (Shanghai Macklin Biochemical Technology Co., Ltd., Shanghai, China) MICs under the experimental conditions used in this study. Bacterial suspensions were adjusted to approximately 2 × 10^5^ CFU/mL in cation-adjusted Mueller–Hinton broth (CAMHB) and exposed to serial concentrations of polymyxin B in 96-well plates. Plates were incubated at 37 °C for 16–18 h, and the MIC was determined by visual inspection as the lowest concentration without visible growth. MIC testing was performed in three independent experiments. For qualitative comparison, serially diluted suspensions were spotted onto LB agar containing polymyxin B and onto antibiotic-free LB agar, followed by overnight incubation at 37 °C; the agar spot assay was also repeated in three independent experiments.

### 2.7. Comparative Disk Diffusion Assay

After incubation for 12 h, bacterial cultures were washed with PBS and adjusted to a 0.5 McFarland turbidity standard. The bacterial suspensions were spread on LB agar plates, and antimicrobial disks were placed on the agar surface. Plates were incubated at 37 °C for 24 h, after which inhibition-zone diameters were measured. Because the assay was performed on LB agar and was not interpreted using clinical breakpoints, the results were used only for comparative analysis among the tested strains.

### 2.8. Transcriptome Sequencing

WT *S.* Typhimurium SL1344 and the Δ*cpxA* mutant were cultured in LB broth containing 0.125 μg/mL polymyxin B, with three independent biological replicates per genotype. The exposure concentration was below all MIC endpoints recorded for these two strains in this study; both genotypes received the same absolute concentration. Differential expression was assessed between the two polymyxin B-exposed genotypes. After growth to logarithmic phase, cells were washed twice with PBS, rapidly frozen in liquid nitrogen, and submitted to Beijing Novogene Bioinformatics Technology Co., Ltd. (Beijing, China) for RNA sequencing. Clean reads were mapped to the selected *Salmonella* reference genome using Bowtie2 version 2.3.4.3, and gene-level read counts were obtained using featureCounts version 1.5.0-p3 in the Subread package. Differential expression analysis was performed using DESeq2 version 1.52.0 in R version 4.6.1 based on raw read counts. Multiple testing was controlled using the Benjamini–Hochberg procedure, and genes with an adjusted *p* value below 0.05 were considered differentially expressed. GO and KEGG over-representation analyses were performed using the FDR-significant gene set, and enrichment *p* values were also adjusted using the Benjamini–Hochberg procedure; adjusted *p* values below 0.05 were considered significant. The transcriptomic data are available in the GEO repository under accession number GSE281216.

### 2.9. Reverse Transcription Quantitative PCR (RT-qPCR)

Bacterial strains were cultured in LB broth containing 0.125 μg/mL polymyxin B with shaking until the logarithmic phase. Total RNA was extracted from the bacterial cultures using TRIzol reagent (Invitrogen, Thermo Fisher Scientific, Carlsbad, CA, USA). Subsequently, cDNA was synthesized from the total RNA via reverse transcription using reagents from Takara. The qPCR reaction mixtures were prepared according to the manufacturer’s instructions for the 2× Universal SYBR qPCR Master Mix (GenOrigin Biotech, Beijing, China; Cat. No. AF05-5). The 16S rRNA gene served as the internal reference for normalization. For each target gene, ΔCt was calculated as Ct_target − Ct_16S, and ΔΔCt was calculated relative to the mean ΔCt of the WT group. Relative expression was calculated using the 2^−ΔΔCt^ method. Four independent biological replicates were analyzed for the WT, Δ*cpxA*, and *cpxA*-complemented strains.

### 2.10. Transmission Electron Microscopy

WT SL1344, the Δ*cpxA* mutant, and the Δ*cpxA*/pBR322-*cpxA* complemented strain were cultured in LB broth containing 0.125 μg/mL polymyxin B. At logarithmic phase, cells were collected, washed with PBS, fixed, deposited onto copper grids, negatively stained, and examined by transmission electron microscopy.

### 2.11. Statistical Analysis

Three independent biological replicates were included in each experiment unless otherwise stated; RT-qPCR experiments included four independent biological replicates per strain. Statistical analyses were performed using GraphPad Prism version 10.6.1. Data are presented as mean ± SEM unless otherwise indicated; RT-qPCR data are presented as mean ± SD. Pairwise comparisons between experimental groups and WT were performed using Student’s *t*-test, with Welch’s correction when variances were unequal. For RT-qPCR, statistical analyses were performed using ΔCt values. For each target gene, overall differences among the WT, Δ*cpxA*, and *cpxA*-complemented strains were assessed using Welch’s one-way ANOVA. Pairwise comparisons were performed using two-tailed Welch’s *t*-tests with Holm–Šídák correction for multiple comparisons. A two-sided *p* value < 0.05 was considered statistically significant.

## 3. Results

The Δ*cpxA* mutant, Δ*cpxA*Δ*arcB* double mutant, and their corresponding plasmid-complemented strains were constructed using the λ-Red homologous recombination system and confirmed by PCR or sequencing before phenotypic analysis.

### 3.1. Phenotypic Characterization of the ΔcpxA and ΔcpxAΔarcB Mutants

**Figure 1:**
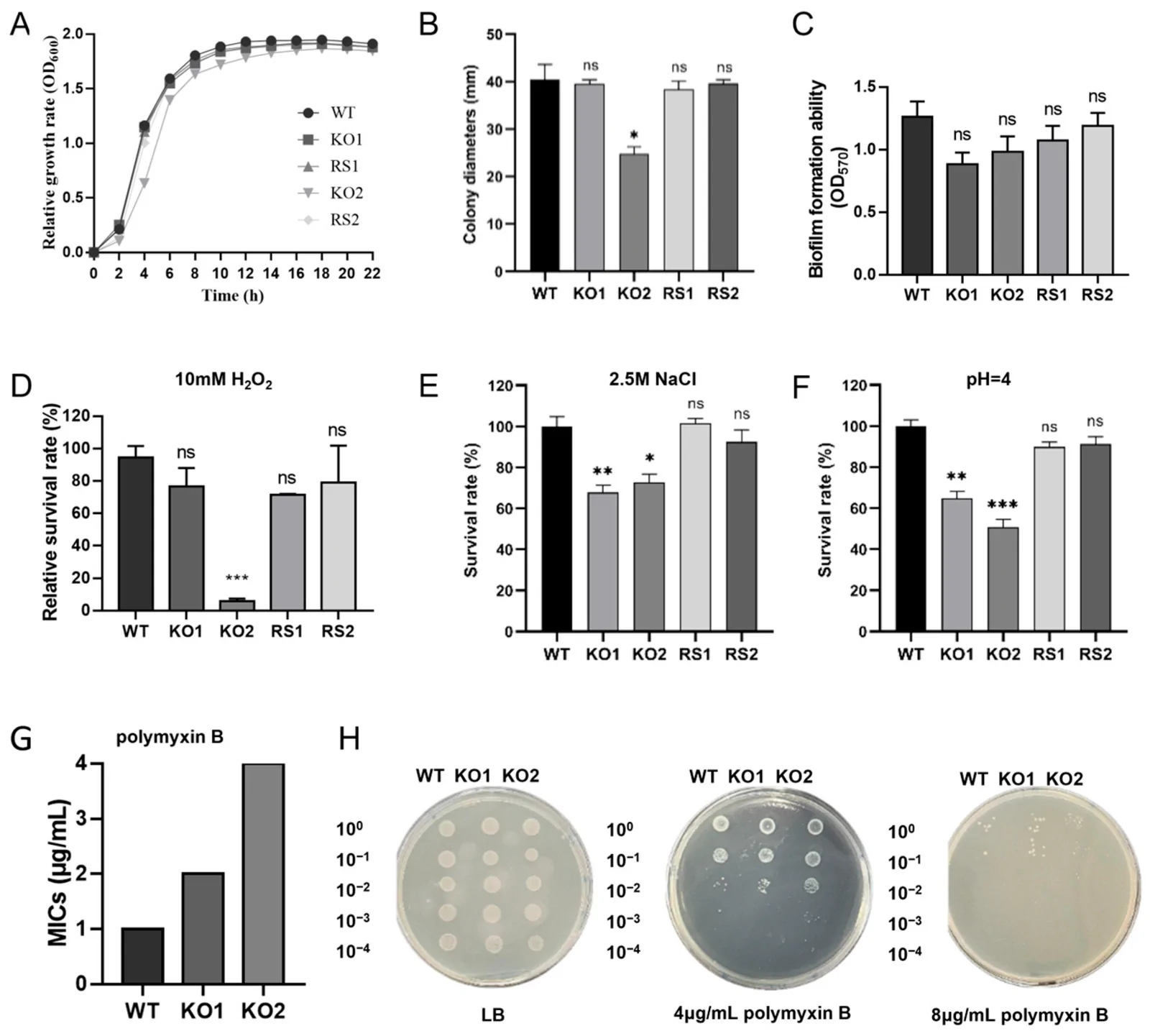
Phenotypic characterization of the Δ*cpxA* and Δ*cpxA*Δ*arcB* strains. (**A**) Growth curves of SL1344 and its derivative strains. (**B**) Swimming motility assay. (**C**) Biofilm formation assay. (**D**) Oxidative-stress survival assay with 10 mM H_2_O_2_. (**E**) Hypertonic-stress survival assay with 2.5 M NaCl. (**F**) Acid-stress survival assay at pH 4.0. (**G**) Summary polymyxin B MICs from three independent experiments. (**H**) Qualitative growth on polymyxin B-containing agar. WT, *S.* Typhimurium SL1344; KO1, SL1344Δ*cpxA*; KO2, SL1344Δ*cpxA*Δ*arcB*; RS1, SL1344Δ*cpxA*/pBR322-*cpxA*; RS2, SL1344Δ*cpxA*Δ*arcB*/pBR322-*cpxA*-*arcB*. Data in panels A–F are presented as mean ± SEM from three independent experiments. Compared with WT, * *p* < 0.05, ** *p* < 0.01, *** *p* < 0.001. ns, not significant.

Growth analysis showed that deletion of *cpxA* did not produce an obvious change in the growth curve compared with WT, whereas the Δ*cpxA*Δ*arcB* double mutant exhibited reduced growth (Figure 1A). Growth of the double-complemented strain was restored toward the WT pattern. Swimming-motility analysis showed no significant difference between WT and the Δ*cpxA* mutant, whereas the Δ*cpxA*Δ*arcB* double mutant exhibited a smaller swimming-zone diameter (Figure 1B). The crystal-violet assay did not detect significant differences in biofilm biomass among the tested strains under the present culture conditions (Figure 1C). Under oxidative-stress conditions, deletion of *cpxA* did not significantly alter survival, whereas the Δ*cpxA*Δ*arcB* double mutant showed reduced survival following exposure to 10 mM H2O2 (Figure 1D). Under hypertonic conditions, both the Δ*cpxA* mutant and the Δ*cpxA*Δ*arcB* double mutant showed lower survival than WT, and survival was restored toward WT levels in the corresponding complemented strains (Figure 1E). Exposure to acidic medium at pH 4.0 also resulted in lower survival of both deletion strains than of WT (Figure 1F). These results indicate that the effects of the mutations differed according to the type of environmental stress.

The reported polymyxin B MICs from three independent broth microdilution experiments were 1, 2, and 4 μg/mL for WT, the Δ*cpxA* mutant, and the Δ*cpxA*Δ*arcB* double mutant, respectively (Figure 1G). The agar spot assay likewise showed greater recovery of the deletion strains than of WT on polymyxin B-containing plates (Figure 1H). Together, the broth and agar assays suggest differences in polymyxin B susceptibility under the tested conditions.

A comparative disk diffusion assay was used to examine inhibition-zone diameters for several antimicrobial agents (Table 1). The Δ*cpxA* mutant showed larger inhibition zones for amikacin, netilmicin, and cephalothin than WT under the tested conditions, and several additional differences were observed in the Δ*cpxA*Δ*arcB* double mutant. Because the assay was conducted on LB agar without clinical interpretive criteria, these differences were considered exploratory and were not used to assign categorical susceptibility or resistance.

### 3.2. Transcriptomic Sequencing Analysis of the ΔcpxA Mutant

To characterize transcriptional changes associated with *cpxA* deletion under polymyxin B exposure, RNA sequencing was performed on WT and Δ*cpxA* cultures exposed to 0.125 μg/mL polymyxin B.

RNA sequencing generated high-quality data for all six libraries. Q30 values ranged from 95.47% to 96.30%, and GC contents ranged from 52.12% to 53.03% (Table 2). Approximately 93.32–95.47% of the clean reads mapped successfully to the selected *Salmonella* reference genome, indicating good sequencing quality and mapping performance.

**Figure 2:**
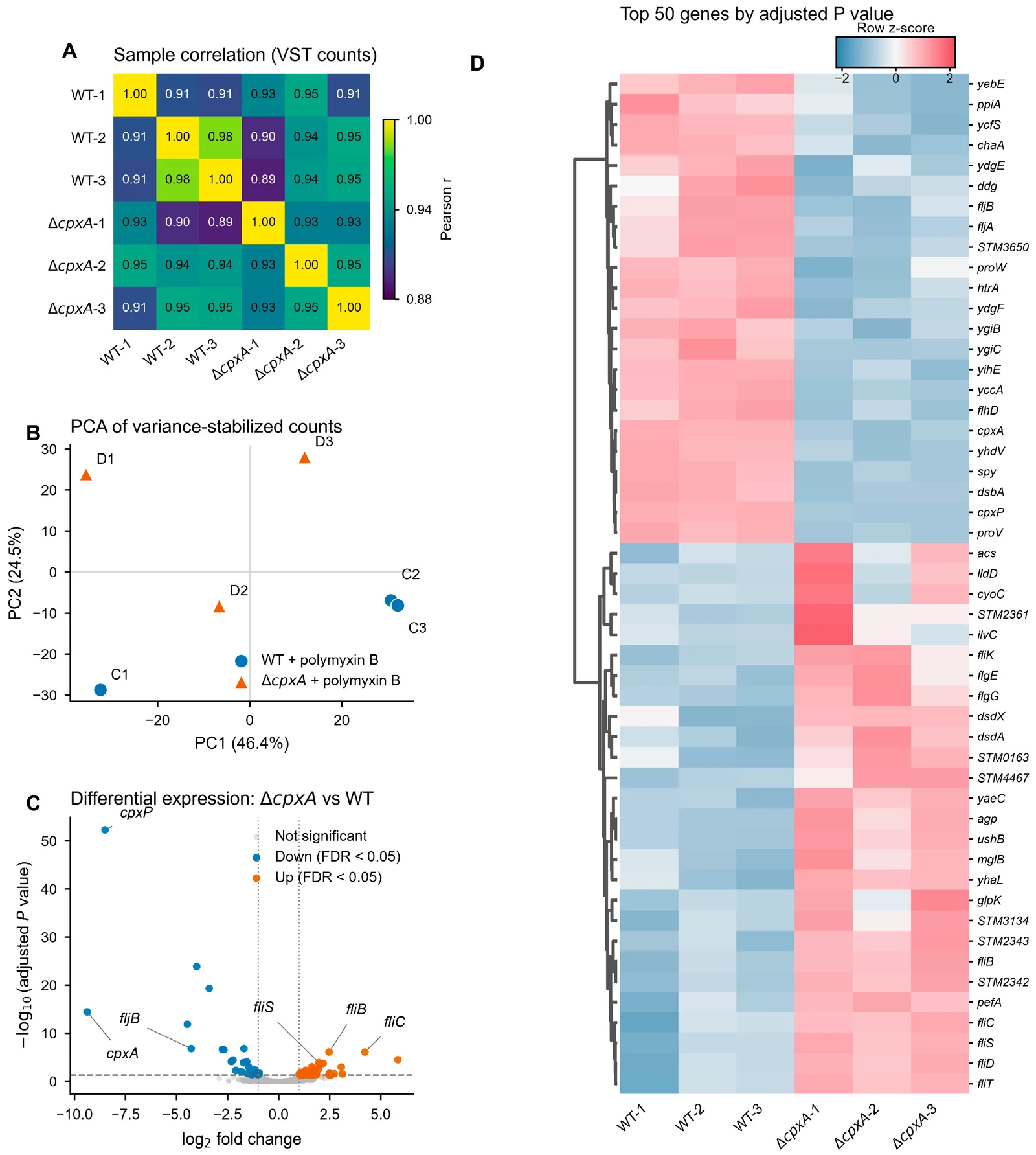
RNA-seq quality assessment and FDR-controlled differential expression under polymyxin B exposure. (**A**) Pairwise Pearson correlations based on variance-stabilized counts. (**B**) Principal-component analysis of WT-PolB and Δ*cpxA*-PolB samples. (**C**) Volcano plot showing differential expression between Δ*cpxA*-PolB and WT-PolB; colored points have Benjamini–Hochberg-adjusted *p* values below 0.05, and vertical dotted lines at log2 fold change ±1 are shown as visual references. (**D**) Clustered heatmap of the 50 genes with the smallest adjusted *p* values; values are row z-scores. Three independent biological replicates were analyzed per genotype.

Pairwise Pearson correlations based on variance-stabilized counts ranged from 0.89 to 1.00 across the six libraries (Figure 2A). Principal-component analysis showed separation between the WT-PolB and Δ*cpxA*-PolB groups, with PC1 and PC2 explaining 46.4% and 24.5% of the variance, respectively (Figure 2B).

DESeq2 analysis with Benjamini–Hochberg correction identified 60 genes with adjusted *p* values below 0.05. Of these, 35 showed higher expression and 25 showed lower expression in the Δ*cpxA*-PolB group than in the WT-PolB group (Figure 2C). A clustered heatmap of the 50 genes with the smallest adjusted *p* values showed distinct group-associated expression patterns across the biological replicates (Figure 2D).

**Figure 3:**
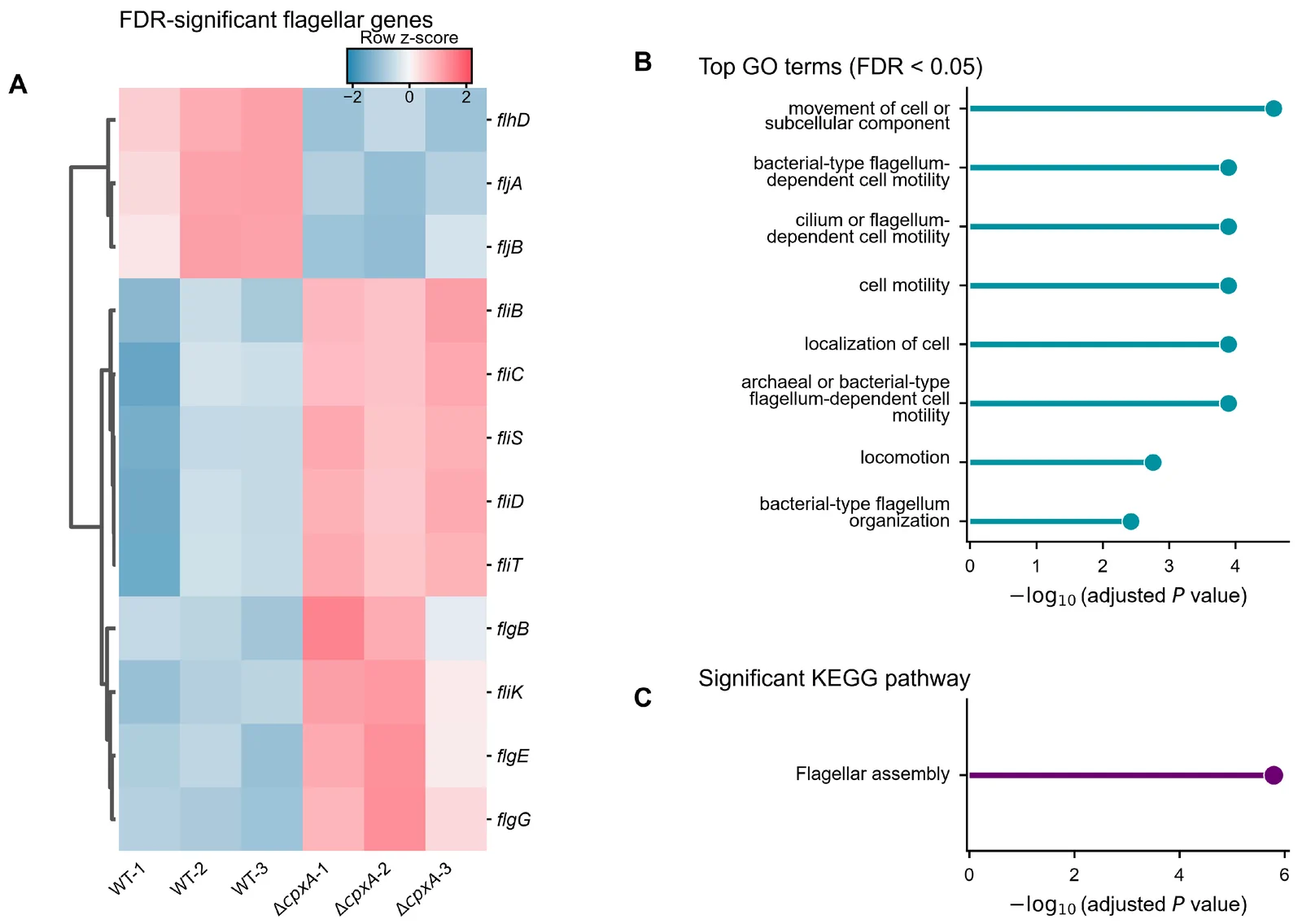
FDR-supported flagellar transcriptional remodeling and functional enrichment. (**A**) Clustered heatmap of the 12 flagellar-associated genes with adjusted *p* values below 0.05; values are row z-scores. (**B**) Eight GO terms significant after Benjamini–Hochberg correction. (**C**) Flagellar assembly, the only KEGG pathway significant after Benjamini–Hochberg correction. Enrichment panels show −log10(adjusted *p* value).

Over-representation analysis of the 60 FDR-significant genes identified eight significant GO terms, primarily related to cell motility and bacterial-type flagellar organization, and one significant KEGG pathway, flagellar assembly (adjusted *p* < 0.05; Figure 3).

#### FDR-Supported Flagellar Transcriptional Remodeling and Functional Enrichment

Among the 60 FDR-significant genes, 12 were assigned to the flagellar gene set. Nine showed higher expression in the Δ*cpxA*-PolB group, whereas *fljA*, *fljB*, and *flhD* showed lower expression. Hierarchical clustering of these genes showed a reproducible group-associated pattern, supporting directionally mixed flagellar transcriptional remodeling rather than uniform upregulation (Figure 3A).

GO analysis of the FDR-significant gene set identified eight significant terms, including movement of a cell or subcellular component, bacterial-type flagellum-dependent cell motility, cell motility, locomotion, and bacterial-type flagellum organization (Figure 3B). KEGG analysis identified flagellar assembly as the only pathway significant after FDR correction, with 10 overlapping genes (Figure 3C).

The FDR-controlled analysis therefore identified selective and directionally mixed transcriptional remodeling of flagellar functions in the Δ*cpxA* strain under polymyxin B exposure. We next assessed selected genes by RT-qPCR and examined extracellular morphology by transmission electron microscopy.

### 3.3. RT-qPCR Assessment of Selected Flagellar-Associated Genes

**Figure 4:**
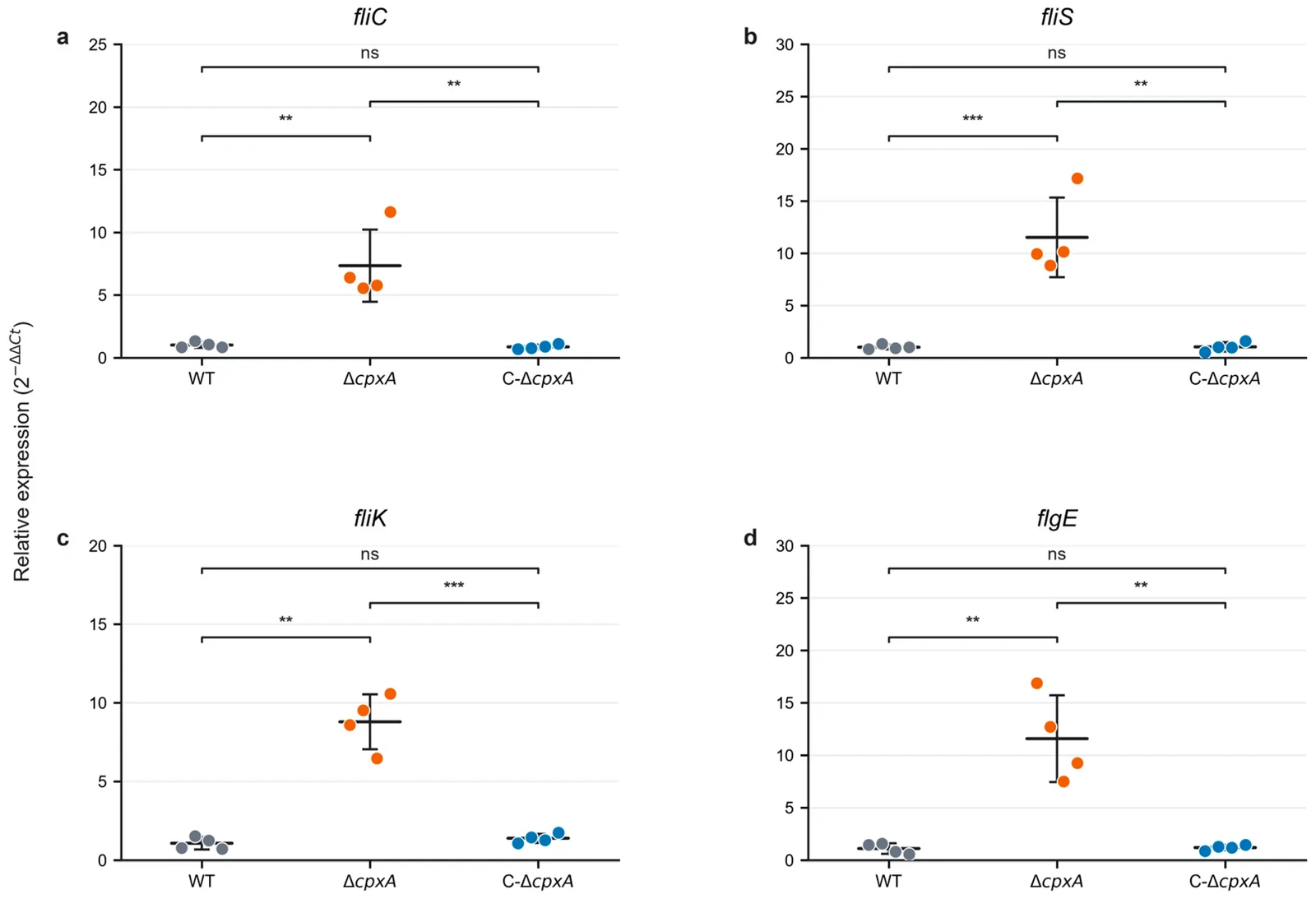
RT-qPCR assessment of selected flagellar-associated genes in the WT, Δ*cpxA*, and *cpxA*-complemented strains under polymyxin B exposure. (**a**) *fliC*; (**b**) *fliS*; (**c**) *fliK*; and (**d**) *flgE*. Relative expression was normalized to 16S rRNA and calculated using the 2^−ΔΔCt^ method, with the mean WT ΔCt for each gene used as the calibrator. Individual points represent four independent biological replicates, and horizontal lines with error bars indicate mean ± SD. Statistical analyses were performed using ΔCt values. Overall differences were assessed using Welch’s one-way ANOVA, followed by pairwise two-tailed Welch’s *t*-tests with Holm–Sidak correction for multiple comparisons. ** *p* < 0.01, *** *p* < 0.001; ns, not significant. C-Δ*cpxA*, *cpxA*-complemented strain.

RT-qPCR was performed to independently assess four flagellar-associated genes selected from the FDR-controlled RNA-seq results. The analyzed genes were *fliC*, *fliS*, *fliK*, and *flgE*, representing flagellin, an export chaperone, hook-length control, and hook structure, respectively. All four genes met the adjusted *p* < 0.05 threshold and showed higher expression in Δ*cpxA* than in WT in the RNA-seq analysis. RT-qPCR likewise showed significantly higher relative expression of all four genes in the Δ*cpxA* strain than in WT (Figure 4). Expression of each gene was significantly lower in the *cpxA*-complemented strain than in the Δ*cpxA* mutant, whereas no significant differences were detected between the complemented strain and WT. Thus, the RT-qPCR assessment was directionally concordant with RNA-seq for all four selected genes and showed restoration toward WT-like expression in the complemented strain.

### 3.4. Qualitative Analysis of Extracellular Filamentous Morphology by Transmission Electron Microscopy

**Figure 5:**
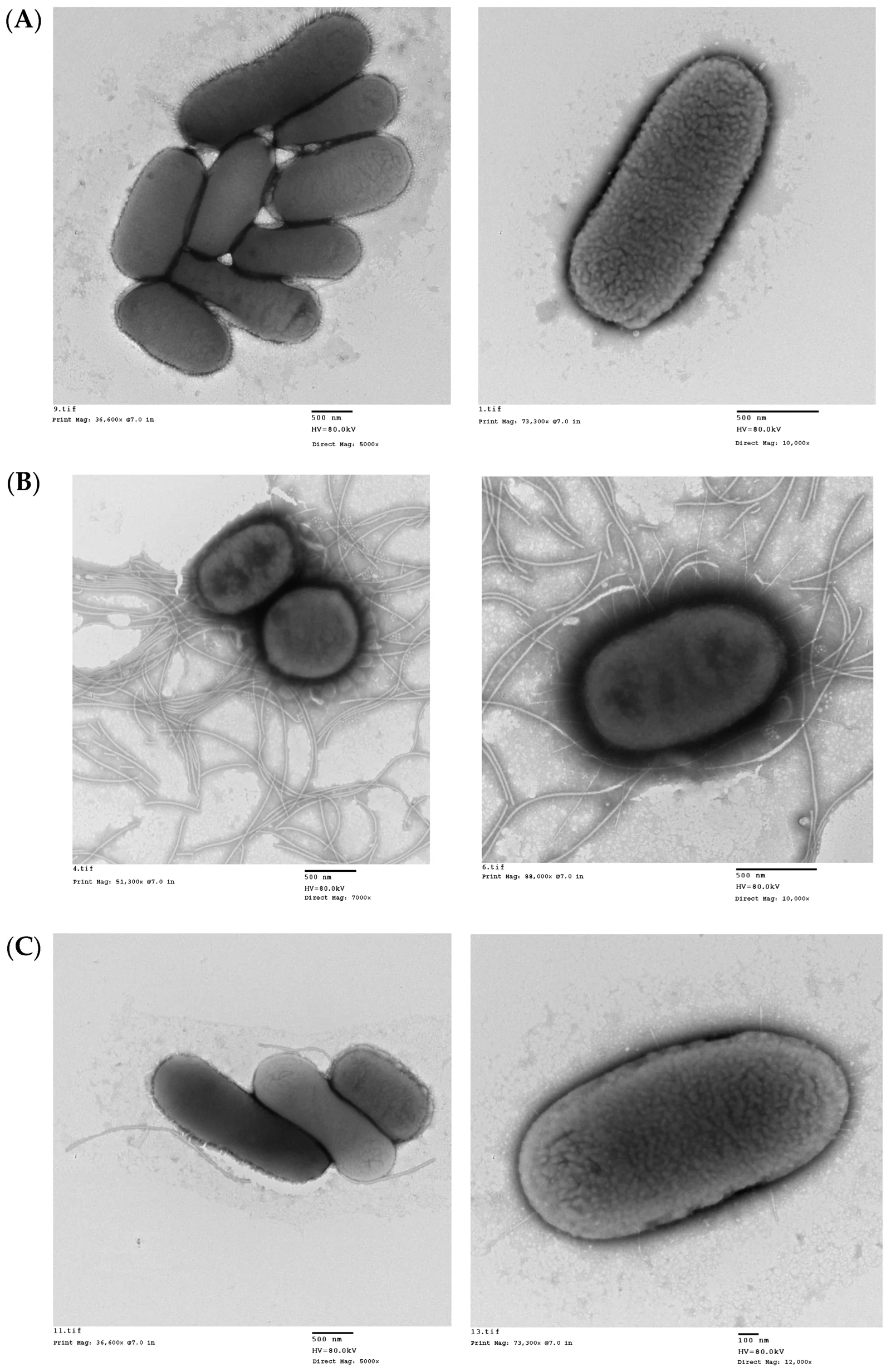
Representative transmission electron micrographs of extracellular filamentous appearance. (**A**) WT *S.* Typhimurium SL1344. (**B**) Δ*cpxA* mutant SL1344Δ*cpxA*. (**C**) Δ*cpxA* complemented strain SL1344Δ*cpxA*/pBR322-*cpxA*. All strains were cultured under polymyxin B exposure before sample preparation. Scale bars are shown in each micrograph.

Transmission electron microscopy was used to examine extracellular filamentous structures under polymyxin B exposure. The representative WT field contained relatively few visible extracellular filaments (Figure 5A), whereas more extensive filamentous structures were visible in the displayed Δ*cpxA* field (Figure 5B). The displayed Δ*cpxA*/pBR322-*cpxA* complemented-strain field appeared more WT-like (Figure 5C).

## 4. Discussion

In the present study, deletion of *cpxA* in *S.* Typhimurium was associated with a one-dilution difference in the reported polymyxin B MIC under the tested conditions. The Δ*cpxA* strain also showed altered tolerance to hypertonic and acidic conditions, whereas deletion of both *cpxA* and *arcB* was associated with broader changes in growth, motility, and stress survival. FDR-controlled RNA-seq identified 60 differentially expressed genes and significant enrichment of flagellar functions, including 12 flagellar-associated genes. RT-qPCR independently supported altered expression of four selected FDR-significant flagellar genes; *fliC*, *fliS*, *fliK*, and *flgE* were directionally concordant with RNA-seq and returned toward WT-like expression in the complemented strain. Representative TEM images provided a qualitative view of extracellular filamentous appearance during polymyxin B exposure. Together, the transcriptional and phenotypic data identify genotype-associated changes under polymyxin B exposure without assigning a causal role to filamentous morphology in the MIC difference.

The regulatory consequences of deleting *cpxA* require cautious interpretation. CpxA possesses both kinase activity toward CpxR and phosphatase activity toward phosphorylated CpxR. Therefore, loss of CpxA does not necessarily represent simple inactivation of the Cpx pathway. Previous work in *S.* Typhimurium showed that deletion of *cpxA* can impair CpxR dephosphorylation and increase the abundance of active CpxR [25]. CpxR may also receive phosphoryl groups from alternative donors under some conditions [26]. Accordingly, the consequences of *cpxA* deletion may involve altered Cpx signaling, but the direction of CpxR activity in the strains examined here was not determined.

CpxR phosphorylation and the relevant regulatory controls were not assessed in this study. CpxR-mediated regulation therefore remains a mechanistic possibility rather than an experimentally assigned signaling state.

The enrichment of flagellar-associated genes is biologically plausible because envelope-stress responses have previously been linked to flagellar regulation in *S.* Typhimurium. Spöring et al. demonstrated that envelope damage and activation of stress-response pathways can affect flagellar biosynthesis through regulation of the FlhD4C2 master regulator [27]. The selective increases in several flagellar-associated transcripts observed in the present Δ*cpxA* strain differ from the repression of flagellar production reported under several other envelope-stress conditions. This difference may reflect the dual kinase-phosphatase function of CpxA, compensatory signaling through CpxR, or differences in the type and duration of the stress stimulus.

The FDR-controlled results also included lower expression of *fljA*, *fljB*, and *flhD* alongside higher expression of nine other flagellar-associated genes. The transcriptomic phenotype is therefore best described as selective flagellar remodeling rather than generalized flagellar overexpression.

The WT and Δ*cpxA* replicate ranges overlapped, placing the one-dilution reported MIC difference in the context of a modest susceptibility shift rather than categorical resistance. The MIC results do not establish altered killing kinetics or bactericidal activity. Its biological basis may involve envelope-related processes. In Gram-negative bacteria, reduced polymyxin susceptibility is commonly associated with lipid A and lipopolysaccharide remodeling, addition of positively charged groups to lipid A, reduced outer-membrane negative charge, altered membrane permeability, and regulation by the PhoPQ and PmrAB two-component systems [23,28,29]. Cpx signaling may influence antimicrobial susceptibility indirectly through broader effects on envelope homeostasis. These established mechanisms provide an important context for interpreting the reported MIC difference observed here and suggest that Cpx-dependent envelope regulation may operate alongside the flagellar-associated changes identified in our study [23,26,27].

Polymyxin B exposure itself may also have contributed to the observed flagellar-associated phenotype. Subinhibitory polymyxin B has been reported to alter cell-associated flagella, motility, and biofilm formation in *Vibrio cholerae* [30].

Accordingly, the RNA-seq results describe genotype-associated expression differences under polymyxin B exposure. The broader sequencing project included an untreated WT group but no untreated mutant group. Thus, the present comparison cannot determine whether these differences were already present before exposure or reflect a differential response to polymyxin B, and it does not estimate a genotype-by-treatment interaction. In addition, a common absolute exposure concentration does not imply an equal fraction of each strain’s reported MIC. Reporting both cultures as logarithmic-phase samples does not by itself demonstrate identical physiological states at harvest.

**Figure 6:**
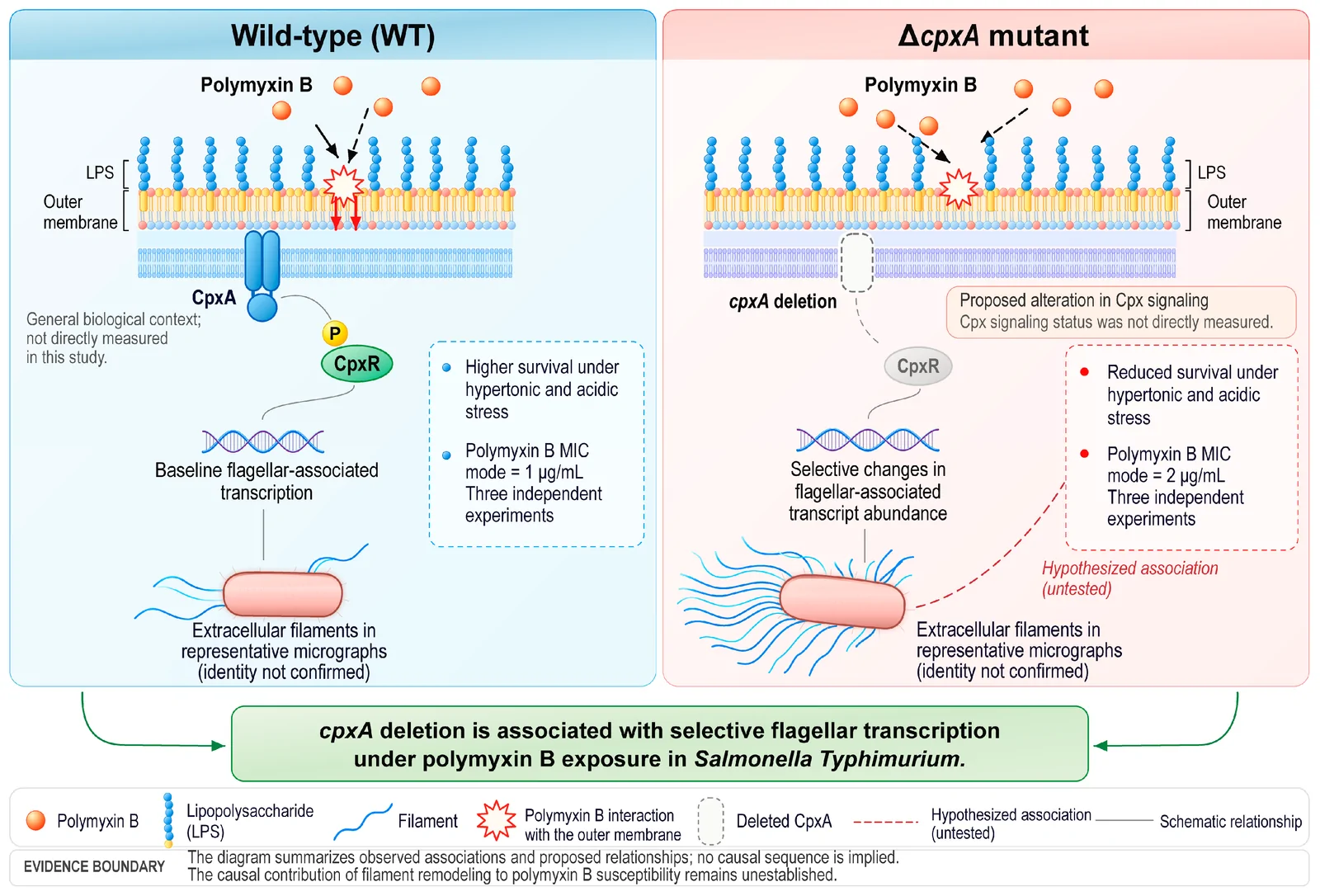
Working hypothesis relating *cpxA* deletion-associated transcriptional changes and polymyxin B susceptibility in *Salmonella* Typhimurium.

Figure 6 summarizes the measured transcriptional and MIC results together with the qualitative TEM observations as a working hypothesis, not a demonstrated causal pathway.

Under polymyxin B exposure, the Δ*cpxA* strain showed selective changes in flagellar-associated transcript abundance and a higher reported polymyxin B MIC than WT, while representative micrographs illustrated differences in the appearance of extracellular filaments of unconfirmed identity. The figure’s ‘Filament’ label is schematic and does not establish the identity of the TEM-visible filaments. CpxA/CpxR signaling and polymyxin B–outer-membrane interaction are shown as biological context; the depicted CpxR phosphorylation states were not measured in these strains. Solid gray lines denote schematic relationships, and the red dashed line denotes a hypothesized, untested association between extracellular filamentous appearance and polymyxin B susceptibility. No causal sequence is implied, and the causal contribution of filament remodeling to polymyxin B susceptibility has not been established.

Despite the higher relative abundance of several flagellar transcripts, the Δ*cpxA* mutant did not exhibit significantly enhanced swimming motility. These observations indicate that the changes in selected transcript levels were not accompanied by enhanced swimming under the tested conditions. Motility depends not only on filament abundance but also on correct flagellar assembly, motor function, chemotactic signaling, and the physiological state of the cell [31]. Similarly, the relationship between flagella and biofilm formation is dependent on the bacterial species, growth stage, surface, and culture conditions [31].

The Δ*cpxA*Δ*arcB* double mutant exhibited more pronounced growth and stress-related phenotypes than the Δ*cpxA* single mutant. ArcAB is a major regulator of respiratory metabolism, redox adaptation, and bacterial fitness [24]. The phenotypic changes observed in the double mutant therefore suggest that simultaneous perturbation of envelope-stress and metabolic-redox regulatory systems may have broader consequences for *Salmonella* physiology. However, no single-mutant comparator for ArcB was included, and direct regulatory measurements and a formal genetic interaction analysis were not performed; therefore, these findings do not establish a direct regulatory or genetic interaction between CpxA and ArcB.

The representative TEM fields showed extracellular filamentous structures, with the complemented-strain field appearing more WT-like. These qualitative observations complement the transcriptional findings; filament identity and strain-level differences in filament number or cell-shape frequency remain to be established.

In conclusion, *cpxA* deletion was associated with selective flagellar-associated transcription under polymyxin B exposure, with the four selected RT-qPCR targets returning toward WT-like expression after complementation. The MIC results showed a one-dilution difference in reported values, while TEM provided representative qualitative images of extracellular filamentous structures. These findings characterize genotype-associated transcriptional and phenotypic responses under the studied condition.

## Figures and Tables

**Table 1:** Comparative inhibition-zone diameters of *S.* Typhimurium and its deletion mutants under the tested disk diffusion conditions.

Antibiotic	Inhibition-Zone Diameter (mm)		
	SL1344	SL1344Δ*cpxA*	SL1344Δ*cpxA*Δ*arcB*
Erythromycin	11.20 ± 0.41	10.13 ± 1.83	13.24 ± 0.49 *
Gentamicin	28.01 ± 1.47	26.33 ± 1.17	31.33 ± 1.65
Chloramphenicol	29.40 ± 0.43	27.16 ± 0.50	32.84 ± 0.84 *
Kanamycin	26.88 ± 0.20	25.81 ± 1.45	28.67 ± 0.39
Tetracycline	23.37 ± 1.23	23.51 ± 0.27	25.62 ± 0.36
Penicillin	17.55 ± 0.93	20.38 ± 1.60	20.05 ± 1.15
Ceftriaxone	27.74 ± 1.30	29.26 ± 0.97	30.67 ± 0.65
Amikacin	25.53 ± 0.70	27.23 ± 0.97 *	29.40 ± 0.64 **
Netilmicin	28.81 ± 0.16	30.07 ± 0.68 *	31.37 ± 0.39 **
Cephalothin	13.77 ± 1.89	18.43 ± 0.95 *	19.66 ± 2.21 *

Data are presented as mean ± SD from three independent experiments. * *p* < 0.05 and ** *p* < 0.01 compared with WT.

**Table 2:** Data quality control.

Sample Name	Raw Reads	Clean Reads	Raw Bases	Clean Bases	Error Rate (%)	Q20 (%)	Q30 (%)	GC Content (%)
A1	7751186	7389894	1.2G	1.1G	0.01	98.66	96.03	52.12
A2	8042464	7464636	1.2G	1.1G	0.01	98.72	96.22	52.29
A3	6517766	6186934	1.0G	0.9G	0.01	98.53	95.66	52.56
B1	6997116	6751964	1.0G	1.0G	0.01	98.59	95.8	52.37
B2	7761466	7397768	1.2G	1.1G	0.01	98.75	96.3	52.72
B3	6838642	6589418	1.0G	1.0G	0.01	98.46	95.47	53.03

A = SL1344-PolB, B = SL1344Δ*cpxA*-PolB.

## Data Availability

The original data presented in the study are openly available in the Gene Expression Omnibus (GEO) under accession number GSE281216.

## Supplementary Materials

The following supporting information can be downloaded at: https://www.mdpi.com/article/10.3390/microorganisms14102237/s1, Table S1: Primers used for strain construction and verification.
