# Supplementary material for: CpxA Deletion Is Associated with Altered Polymyxin B Susceptibility and Flagellar-Associated Transcriptional Remodeling in Salmonella Typhimurium
Source: Microorganisms. 2026 Oct 3;14(10):2237. doi: 10.3390/microorganisms14102237 (PMC13707502; doi:10.3390/microorganisms14102237)
Supplement: Supplementary file 1 [file microorganisms-14-02237-s001.zip › microorganisms-4593122-supplementary.pdf]

Table S1. Primers used for strain construction and verification.

Primer ID	Sequence (5′–3′)	Expected amplicon length (bp)
P1	GCCAGGTGGTTTCCCG	216
P2	CAGCTCCAGCCTACACGCGCGGTTAAACTTC	216
P3	GAAGTTTAACCGCGCGTGTAGGCTGGAGCTG	1512
P4	TAGGTTCGCTTGTACAGCGGCATATGAATATCCTCCTTA	1512
P5	TAAGGAGGATATTCATATGCCGCTGTACAAGCGAACCTA	207
P6	CCGTTTGCCGGTAATCGCTTT	207
P7	GATGATTCGTCATACGCCGC	301
P8	CAGCTCCAGCCTACACATAGAGGATTCCTTCACCACAACTT	301
P9	AAGTTGTGGTGAAGGAATCCTCTATGTGTAGGCTGGAGCTG	1522
P10	GCACCCCGGTCAAACCGGGGCATATGAATATCCTCCTTA	1522
P11	TAAGGAGGATATTCATATGCCCCGGTTTGACCGGGGTGC	310
P12	GCCTCATGAATTTCGGTGCCGT	310
P13	TTTGACAGCTTATCATCGATAATGGTTTCCGCTTCATGAT	1440
P14	CACGATGCGTCCGGCGTAGAGTTAGGTTCGCTTGTACAG	1440
P15	TTTGACAGCTTATCATCGATAATGAAGCAAATTCGTCTG	2911
P16	CTTCCTATCATGAAGCGGAAACCATTCATTTTTAGTGGCTTTTG	2911
P17	GGTGGCAAAAGCCACTAAAAAATGAATGGTTTCCGCTTCATGATA	1434
P18	CACGATGCGTCCGGCGTAGAGTTAGGTTCGCTTGTACAGCG	1434
P19	CAGCGTTGAGGCCATAACAG	WT: 2536
P20	CGCTACAAGTGGGTGAAGAAGG	cpxA deletion strain: 1256
P21	CGGTGCCCTGAATGAACTGC	471
P22	CGGCCACAGTCGATGAATCC	471
P23	CGAACTGGACGACTACACG	WT: 2865
P24	TGCAACCGACTCAAGCC	arcB deletion strain: 591
P25	CTCATGTTTGACAGCTTATC	cpxA-complemented strain: 1664
P26	CAGCCCAGTAGTAGGTTGAG	ArcB-cpxA-complemented strain: 4243
